# Lack of Contribution of p66shc to Pressure Overload-Induced Right Heart Hypertrophy

**DOI:** 10.3390/ijms21249339

**Published:** 2020-12-08

**Authors:** Christine Hirschhäuser, Akylbek Sydykov, Annemarie Wolf, Azadeh Esfandiary, Julia Bornbaum, Hanna Sarah Kutsche, Kerstin Boengler, Natascha Sommer, Rolf Schreckenberg, Klaus-Dieter Schlüter, Norbert Weissmann, Ralph Schermuly, Rainer Schulz

**Affiliations:** 1 Physiologisches Institut, Justus-Liebig-Universität, 35392 Gießen, Germany; Annemarie.Wolf@Physiologie.med.uni-giessen.de (A.W.); Julia.Bornbaum@physiologie.med.uni-giessen.de (J.B.); Hanna.Kutsche@physiologie.med.uni-giessen.de (H.S.K.); Kerstin.Boengler@physiologie.med.uni-giessen.de (K.B.); Rolf.Schreckenberg@physiologie.med.uni-giessen.de (R.S.); Klaus-Dieter.Schlueter@physiologie.med.uni-giessen.de (K.-D.S.); Rainer.Schulz@physiologie.med.uni-giessen.de (R.S.); 2 Excellence Cluster Cardiopulmonary System (ECCPS), Justus-Liebig-Universität, 35392 Gießen, Germany; Akylbek.Sydykov@innere.med.uni-giessen.de (A.S.); Azadeh.Esfandiary@yahoo.com (A.E.); Natascha.Sommer@innere.med.uni-giessen.de (N.S.); Norbert.Weissmann@innere.med.uni-giessen.de (N.W.); Ralph.Schermuly@innere.med.uni-giessen.de (R.S.)

**Keywords:** reactive oxygen species, cardiac hypertrophy, p66shc, heart failure

## Abstract

The leading cause of death in pulmonary arterial hypertension (PAH) is right ventricular (RV) failure (RVF). Reactive oxygen species (ROS) have been suggested to play a role in the development of RV hypertrophy (RVH) and the transition to RVF. The hydrogen peroxide-generating protein p66shc has been associated with left ventricular (LV) hypertrophy but its role in RVH is unclear. The purpose of this study was to determine whether genetic deletion of p66shc affects the development and/or progression of RVH and RVF in the pulmonary artery banding (PAB) model of RV pressure overload. The impact of p66shc on mitochondrial ROS formation, RV cardiomyocyte function, as well as on RV morphology and function were studied three weeks after PAB or sham operation. PAB in wild type mice did not affect mitochondrial ROS production or RV cardiomyocyte function, but induced RVH and impaired cardiac function. Genetic deletion of p66shc did also not alter basal mitochondrial ROS production or RV cardiomyocyte function, but impaired RV cardiomyocyte shortening was observed following PAB. The development of RVH and RVF following PAB was not affected by p66shc deletion. Thus, our data suggest that p66shc-derived ROS are not involved in the development and progression of RVH or RVF in PAH.

## 1. Introduction

Cardiac hypertrophy occurs as a result of a variety of heart diseases, including pulmonary arterial hypertension (PAH). PAH increases right ventricular (RV) afterload, inducing—if prolonged—RV hypertrophy (RVH). In the compensated state, RVH reduces RV wall stress, and thus energy demand, and helps to maintain cardiac output. However, sustained pressure overload progresses into pathological remodeling of the RV, leading to dilation and finally culminating in right ventricular failure (RVF) [1].

Reactive oxygen species (ROS) and their excessive production (oxidative stress) have been suggested to play a role in RVH and remodeling [2,3]. While smaller amounts of ROS act as signaling molecules and contribute to cardiac hypertrophy, oxidative stress contributes to the transition of adaptive to maladaptive cardiac hypertrophy (maladaptive remodeling) and the development of RVF [4,5,6].

Oxidative stress can induce cardiac dysfunction by activating p38 mitogen activated protein (MAP) kinase pathways, by oxidative modification of contractile proteins, by disturbing metabolism and the intracellular ion homeostasis, by damaging mitochondria, or by inducing cell death [6,7,8,9,10]. Therefore, ROS defense strategies are necessary to promote cell survival and maintain cellular function. Indeed, antioxidants prevent the development of heart failure in several animal studies [11,12,13].

In the pressure overloaded RV, elevated ROS quantities mainly originate from nicotinamide adenine dinucleotide phosphate (NADPH) oxidases and mitochondria [6]. Within the mitochondria, ROS can be formed by different complexes of the electron transport chain (ETC) [6]. Increased ROS release from complex II is suggested to be important for the transition from RVH to RVF. However, besides the ETC, other enzymes also contribute to ROS formation under pathophysiological conditions (for review, see [5]).

An additional protein contributing to mitochondrial ROS formation is the adaptor protein p66shc. p66shc—together with p46shc and p52shc—is one of three isoforms encoded by the Shc1 gene. The three isoforms share a Src-homology domain (SH2), a collagen-homology region (CH1), and a phosphotyrosine-binding domain (PTB). However, due to alternative promotor usage, p66shc contains an additional N-terminal CH region (CH2), which includes the important phosphorylation site serine 36 (S36). Under basal conditions, p66shc is predominantly located in the cytosol, but becomes phosphorylated at S36 by kinases that are activated in response to various stress signals [14,15]. As a result, p66shc translocates to the mitochondrial intermembrane space where it induces ROS formation. Mitochondrial p66shc oxidizes cytochrome c and thereby catalyzes the reduction of oxygen to hydrogen peroxide [16]. p66shc phosphorylation also negatively regulates the transcription of ROS scavenging enzymes, such as manganese superoxide dismutase (MnSOD) and catalase [17,18]. Furthermore, p66shc triggers cytosolic ROS formation by promoting the formation of an active NADPH oxidase complex [19]. Accordingly, reduced levels of ROS in response to various stimuli are demonstrated in different p66shc-deficient cell types and organs, including endothelial cells [20,21] and myocardial tissue [22]. Moreover, the reduced production of ROS in p66shc-deficients cells is associated with resistance to apoptosis [23].

p66shc protein amounts are high in neonatal, but low in adult cardiomyocytes. This is of relevance since the molecular adaption of the heart to pressure overload is associated with the reactivation of fetal genes. Indeed, induction of p66shc protein expression in the left ventricle (LV) upon hypertrophic stimuli has been reported in vitro [24] and in vivo [25]. Hypertrophic stimuli also increase p66shc S36-phosphorylation [24]. Moreover, the genetic deletion of p66shc prevents LV from hypertrophy and apoptotic cell death [26]. However, there is growing evidence that the LV and the RV differ in their ability to respond to pressure overload [27]. In fact, the RV has a reduced oxidative defense capacity, which in turn could promote oxidative stress and thus the transition from RVH to RVF. So far, no studies have been conducted to investigate the role of p66shc in the adaptation of the RV to pressure overload. Therefore, the aim of the current study was to investigate the impact of p66shc-mediated ROS formation on RV function and structure upon pulmonary artery banding (PAB) in mice.

## 2. Results

At the beginning of this study the genotype of p66shc knockout (p66KO) mice was confirmed. Western blot analysis was performed for p66shc in LV tissue samples of wild type (WT) and p66KO mice. Ponceau S staining served to demonstrate equal protein loading. A 66-kDa band, which corresponds to the molecular weight of p66shc, was detected in the LV of WT, but not of p66KO mice (Figure S1).

**Figure 1:**
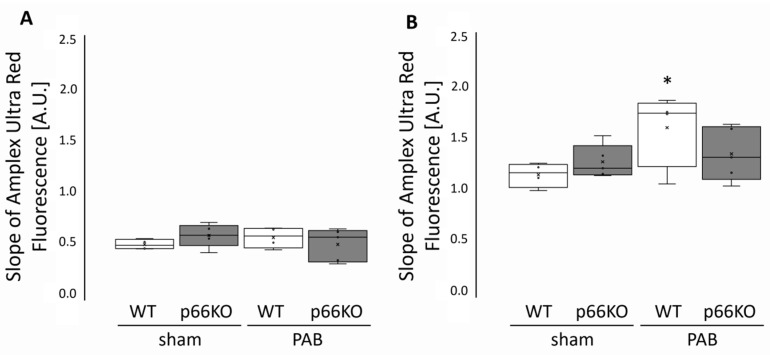
ROS formation in mitochondria isolated from mice three weeks after pulmonary artery banding (PAB) or sham surgery. (**A**) Basal and (**B**) rotenone-stimulated reactive oxygen species (ROS) production in right ventricular (RV) mitochondria. ROS formation was measured as the slope of Amplex Ultra Red fluorescence over time in arbitrary units (A.U.). Data are given for wild type (WT) and p66shc knockout (p66KO) mice three weeks after sham surgery (*n* = 4 and *n* = 5, respectively) and for WT and p66KO mice three weeks after PAB (*n* = 6 and *n* = 5, respectively). Data are expressed as 25%, 50%, and 75% quartiles with whiskers representing the total range. The dots represent the individual data points. *: *p* < 0.05 vs. WT sham as analyzed by two-way ANOVA.

ROS formation was measured in mitochondria isolated from RV tissue of WT and p66KO mice three weeks after PAB or sham operation using Amplex Ultra Red fluorescence. ROS production was characterized without (basal) or with the complex I inhibitor rotenone (Figure 1). Basal ROS formation did not differ between both genotypes, neither after sham operation nor after PAB surgery. Treatment of isolated mitochondria with rotenone increased ROS production in all groups. However, rotenone-induced ROS formation was significantly higher in RV mitochondria isolated from banded WT mice than in sham-operated WT mice. This effect was not observed in mitochondria isolated from banded p66KO RVs.

Subsequently, we investigated whether the deletion of p66shc impacts the function of RV cardiac myocytes following three weeks of pressure overload or sham operation. The diastolic cell length (Ldiast), the load-free cell shortening (quantified as percent shortening amplitude normalized to the diastolic cell length of individual cells, dL/L) as well as the contraction- and relaxation velocities were analyzed (Table 1). We did not detect differences in Ldiast of RV myocytes, neither between WT or p66KO cells nor following PAB or sham operation. Similarly, contraction and relaxation velocities were not affected following PAB in WT and p66KO cardiomyocytes. However, p66KO cardiomyocytes displayed a significant reduction of dL/L after three weeks of PAB compared to WT cells (Table 1).

The hypertrophy and function of the RV were analyzed in WT and p66KO mice by hemodynamic measurements and echocardiography three weeks after PAB or sham operation. Body weight (BW) and heart rate (HR) were not different between WT and p66KO mice following PAB or sham operation (Table 2).

**Figure 2:**
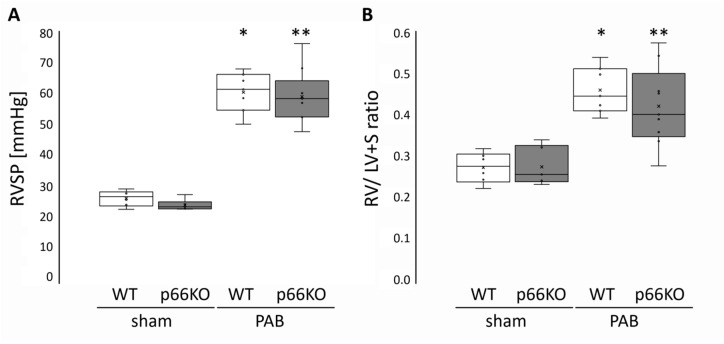
Right ventricular systolic pressure (RVSP) and hypertrophy in mice three weeks after pulmonary artery banding (PAB) or sham surgery. (**A**) RVSP (in mmHg) and (**B**) right ventricular weight normalized to the left ventricle and septum weight (RV/LV + S ratio). Data are given for wild type (WT) and p66shc knockout (p66KO) mice three weeks after sham surgery (*n* = 6 each group) and for WT and p66KO mice three weeks after PAB *(n* = 7 and *n* = 9, respectively). Data are expressed as 25%, 50%, and 75% quartiles with whiskers representing the total range. The dots represent the individual data points. *: *p* < 0.05 vs. WT sham and **: *p* < 0.05 vs. p66KO sham as analyzed by two-way ANOVA.

RV systolic pressure (RVSP) increased similarly in WT and p66KO mice following PAB (Figure 2A) whereas systemic blood pressure (SAP) remained constant (Table 2). WT and p66KO mice developed RV hypertrophy and dilatation of comparable degree after PAB as indicated by a similar increase in the ratio of the RV weight over that of the LV and septum (RV/LV+ S ratio) (Figure 2B) and the ratio of the RV to BW (RV/BW), as well as by enhanced RV wall thickness (RVWT) and RV inner diameter (RVID) (Table 3). LV mass (in mg/g BW) was not affected by PAB surgery (WT PAB: 1.9 ± 0.10, *n* = 7 vs. WT sham: 2.0 ± 0.13, *n* = 6, *p* = ns, and p66KO PAB: 1.9 ± 0.26, *n* = 9 vs. p66KO sham: 1.8 ± 0.21, *n* = 6, *p* = ns, respectively) and did not differ between WT and p66KO mice.

**Figure 3:**
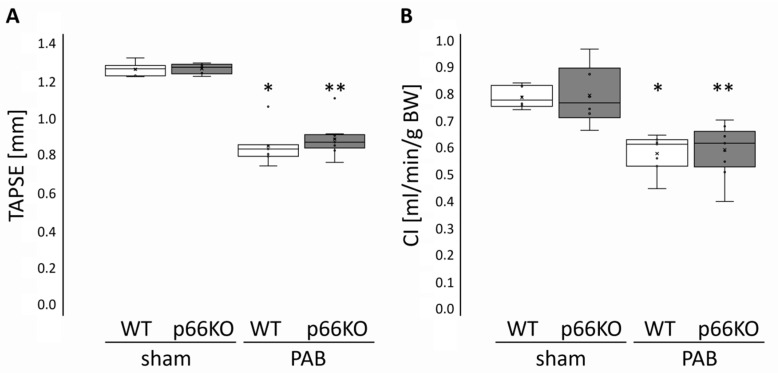
Cardiac function in mice three weeks after pulmonary artery banding (PAB) or sham surgery. (**A**) Tricuspid annular plane systolic excursion (TAPSE, in mm) and (**B**) cardiac index (CI, in ml/min/g BW). Data are given for wild type (WT) and p66shc knockout (p66KO) mice three weeks after sham surgery (*n* = 6 each group) and for WT and p66KO mice three weeks after PAB (*n* = 7 and *n* = 9, respectively). Data are expressed as 25%, 50%, and 75% quartiles with whiskers representing the total range. The dots represent the individual data points. *: *p* < 0.05 vs. WT sham and **: *p* < 0.05 vs. p66KO sham as analyzed by two-way ANOVA.

Tricuspid annular plane systolic excursion (TAPSE)—a commonly used parameter of RV function—as well as cardiac index (CI) were determined by echocardiography. Both parameters decreased similarly in WT and in p66KO mice three weeks after PAB compared to sham operated animals (Figure 3).

Prior to the analysis of ROS formation and cardiomyocyte function, RV weight and RV function were also characterized by echocardiography before the isolation of mitochondria or cardiomyocytes, respectively, in order to validate the extent of hypertrophy and the impairment of RV function three weeks after PAB (Tables S1 and S2). Mice in these study groups developed a similar increase in RVWT and showed reduced TAPSE and CI three weeks after PAB, again with no differences between WT and p66KO mice.

To achieve a higher n-value for the statistical analysis of putative effects of the p66shc deletion on the RV, data obtained by echocardiography were pooled from the animals of all study groups. However, no differences were found in the development of RVH and the impairment of RV function between WT and p66KO mice three weeks after PAB or sham operation (Table S3).

Using echocardiography, accurate measurements of LV structure and function following PAB are not possible due to significant impairment of LV shape caused by PAB-induced RV hypertrophy and dilatation [28]. Therefore, LV structure and function were measured in sham operated WT and p66KO mice only (Table S4). WT and p66KO mice displayed comparable LV mass, thickness of the interventricular septum (IVS) and LV posterior wall (LVPW). However, LV inner diameter in the systole (LVIDs) but not in the diastole (LVIDd) was increased, whereas fractional shortening (FS) was decreased in p66KO mice compared to WT mice without affecting CI, confirming our previous data [29].

## 3. Discussion

The current study aims to clarify the role of p66shc in the development of RVH and RVF. The main findings of this study are: (1) that RV pressure overload did not increase basal mitochondrial ROS formation, but increased stress-induced ROS formation in a p66shc-dependent manner; (2) that p66shc-deficient RV myocytes displayed an impaired load-free cell shortening following pressure overload; and (3) that the development of RVH and RVF upon pressure overload was not affected by the genetic deletion of p66shc.

p66shc contributes to ROS formation by the oxidation of cytochrome c and the catalyzed reduction of oxygen to hydrogen peroxide (H_2_O_2_) [16]. In the present study, H_2_O_2_ generation was similar between RV mitochondria isolated from sham operated WT and p66KO mice. This finding is in line with previous data showing that lipid peroxidation and tropomyosin oxidation as markers of oxidative stress were comparable in isolated hearts of both genotypes [22]. However, others describe reduced ROS levels in p66shc-deficient cells [30,31,32].

Also, similar ROS formation was detected in WT compared to p66KO mitochondria following three weeks of PAB. There is evidence that mitochondrial ROS production is decreased in the compensated state of RVH whereas the transition from RVH to RVF is associated with oxidative stress [33]. The onset and timing of changes in ROS levels during RVH and RVF are not clearly defined and differ within models and species. In a recent study using monocrotaline (MCT)-induced PAH in rats, H_2_O_2_ concentration in RV homogenates increased after one week following MCT–induced pressure overload but returned to control values after two weeks and three weeks [34]. However, increased ROS production due to enhanced activities of mitochondrial complex II and NADPH oxidase were detected in RV tissue homogenates and isolated mitochondria 25 days after MCT-injection [6]. In the PAB model H_2_O_2_, but not superoxide (O_2_^−^) formation increases in the acute phase, i.e., 6 h after surgery and returns to control values after 24 h, suggesting that H_2_O_2_ is the major ROS acutely produced. NADPH oxidase inhibitor diphenyleneiodonium chloride diminished this increase and therefore suggests that increased H_2_O_2_ production following acute PAB is related to NADPH oxidase 4 activity [35]. However, a robust increase in O_2_^−^ was observed after three and four weeks of chronic pressure overload induced by PAB [36,37].

In the presence of rotenone ROS formation was significantly higher in WT mitochondria after PAB compared to mitochondria isolated from sham operated animals. Interestingly, this was not true for mitochondria isolated from p66KO RV. Rotenone increases ROS formation by inhibiting the electron transfer from complex I to ubiquinone [38]. The PAB-mediated increase in ROS production following rotenone administration may indicate p66shc activity in WT mitochondria, since p66shc was absent in p66KO mitochondria. Mitochondrial p66shc is associated within a high molecular inhibitory complex under basal conditions but dissociates upon oxidative stress [39]. Thus, rotenone-induced ROS formation may represent a sufficient stimulus to acutely activate mitochondrial p66shc contributing to further ROS formation in WT mitochondria but not in mitochondria lacking p66shc. Alternatively, the missing increase in PAB-mediated ROS production in p66KO mitochondria may be the consequence of reduced ROS defense capacity due to p66shc-mediated downregulation of ROS-scavenging proteins in WT but not in p66KO animals [40]. In fact, p66shc-deficient mice displayed a stronger resistance to oxidative stress [23].

In addition to mitochondrial p66shc also cytosolic p66shc contributes to ROS formation [19]. This in turn can directly reduce cardiomyocyte function by oxidative modification of contractile proteins [41]. Therefore, we investigated whether p66shc deletion regulates the function of RV myocytes upon pressure overload. It is accepted that a functional impact of hypertrophy is an increased contractility, a common feature of patients with PAH [42] and of animals with pressure overload [43]. In our study, RV myocytes from WT hearts showed a tendency towards increased contractility, as evidenced by the increase in contraction velocity, relaxation velocity and load-free cell shortening. In a previously published study, we have already described improved function of WT RV myocytes following three weeks of PAB [44]. However, four weeks after PAB RV cardiomyocytes are significantly impaired in their function, pointing towards decompensation of the RV at this time point [36].

p66shc-deficient and WT RV myocytes did not differ in their diastolic cell length, contraction, and relaxation velocities. However, load-free cell shortening following PAB was reduced in RV myocytes isolated from p66KO hearts compared to WT cardiomyocytes. Redox-sensitive amino acids within the cardiac ryanodine receptor (RyR2) and the sarcoplasmic reticulum calcium ATPase (SERCA) imply an association between the electromechanical coupling system and oxidative stress [45]. However, since mitochondrial ROS formation did not differ between both genotypes following PAB, it is unlikely that the reduced load-free cell shortening mediated by p66shc deletion was related to mitochondrial ROS.

LV structure and systolic function were characterized by echocardiography in WT and p66KO mice three weeks after sham surgery. LV mass, SAP and HR were similar between the genotypes, which confirm already published data by Graiani and coworkers [26,46]. Surprisingly, only few studies investigated basal LV dimensions and LV volume in p66KO mice with controversial results. One study reported LV dimensions comparable with WT mice along with decreased LV chamber volume in p66KO mice [26]. Yet another study did not reveal any effect of the p66shc deficiency on LV dimensions and volume [47], while our own previous analysis in WT and p66KO mice confirmed the data of the present study [29]. The reasons for the discrepancy are unclear. However, it is well known that tissue fixation affects significantly the cardiac parameters [48]. We believe that the different methodological approaches (measurements in formalin-fixed hearts [26,47] versus echocardiography-derived measurements (present study, [29,46]) may account for the discrepancy. Further studies on larger number of animals are necessary to clarify this issue.

It is described that genetic deletion of p66shc prevents the LV from angiotensin II (Ang II)-induced hypertrophy and apoptotic cell death [26]. In the aforementioned study the authors administered Ang II chronically via osmotic pumps, therefore, the drug had the potential to act on both ventricles. However, the RV did not develop hypertrophy indicating that the signaling pathways leading to RVH may be different from that leading to LV hypertrophy (LVH). Also, hypertrophic pathways induced by transverse aortic constriction (TAC) in the LV [49], were not activated in the RV following PAB [50]. Differences in the hypertrophic signaling pathways in LV and RV tissue would explain why common drugs to treat LV failure such as Ang II antagonists do not improve heart function in patients with RVF [51,52]. In the present study we investigated whether p66shc, which contributes to the development of LVH, also plays a role in RVH. In accordance with the previously published experimental studies [44,53], we demonstrated that chronic pressure overload induced by PAB resulted in RV hypertrophy, dilatation and impaired RV function. However, our data did not indicate differences between WT and p66KO hearts regarding hemodynamic, functional, and morphological parameters suggesting that p66shc deletion per se had no impact on the development of RV hypertrophy and function upon pressure overload. Similarly, our recent work demonstrated that p66shc deletion does not influence cardiac function in hypoxia-induced pulmonary hypertension [54]. Our previous data also exclude a contribution for p66shc in myocardial ischemia/reperfusion injury and the protection from it by ischemic preconditioning, both conditions in which ROS play important roles [55]. Taken together, our data show that p66shc does not contribute to the development and progression of pressure overload-induced RVH three weeks after PAB. Since the RV is derived from a different embryonic origin than the LV and there are indications that the pro-hypertrophic signaling pathways differ, at least in part, between both ventricles, our findings may suggest that p66shc is either not activated at all in the RV upon pressure overload or its activation occurs at a time point when p66shc is not of relevance for the fate of the RV.

## 4. Study Limitations

The development of RVH and RVF upon PAB is a continuous process that can activate different signaling molecules in a time-dependent manner. In our study, we analyzed only one time point, i.e., three weeks after PAB or sham operation, where p66shc fails to influence RV hypertrophy and function. The investigation of the analyzed parameters at different time spans may reveal other results regarding the role of p66shc in RVH and RVF. In the present study we could not support or rule out p66shc activation—as indicated by phosphorylation at S36 or by the mitochondrial translocation of the protein—after PAB surgery in WT mice. Also, putative effects of p66shc on mitochondrial function, i.e., oxygen consumption, expression, and activities of complexes of the ETC or of biogenetic factors were not addressed. Recent work provides evidence that p66shc expression and activation positively correlates with elevated mitochondrial oxidative phosphorylation in the central nervous system [56] and in cultured mouse embryos [57]. Cardiac fibrosis is a major factor in heart remodeling and the development of hypertrophy influences cardiac function. However, the present study does not address whether or not p66shc is implicated in the development of RV cardiomyocyte hypertrophy and fibrosis following PAB. Therefore, the role of p66shc on RV hypertrophy and fibrosis after RV pressure overload remains unclear.

## 5. Materials and Methods

### 5.1. Animals and Ethical Concerns

The present study conforms to the Guide for the Care and Use of Laboratory Animals published by the US National Institutes of Health (NIH publication No. 85–23, revised 1996) and was approved by the “Regierungspräsidium Gießen” (GI 20/10 Nr. 40/2011 (permission granted 16th May 2012); GI 20/1 Nr. 91/2017 (permission granted 12th February 2018)). In the study, 15–43 weeks old male C57Bl6/J mice (25–30 g, Janvier, Le Genest-Saint-Isles, France) and p66shc knockout (p66KO) mice were used. The p66KO mice were generated in the laboratory at the European Institute of Oncology (Milan, Italy) by Marco Giorgio [23]. Mice were kept in dark/light cycles of 12 h each and had free access to standard chow and drinking water.

### 5.2. Pulmonary Artery Banding (PAB) In Vivo

**Figure 4:**
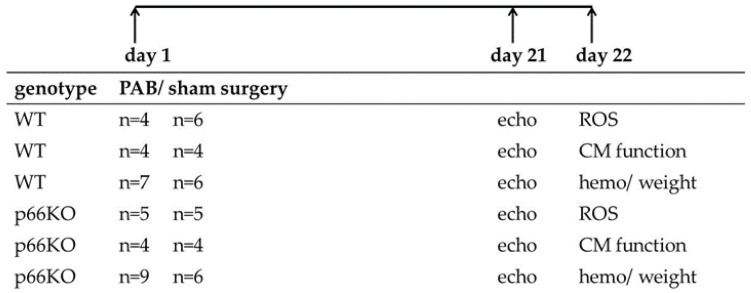
Design of the study. In total 31 wild type (WT) and 33 p66shc knockout (p66KO) mice were subjected to pulmonary artery banding (PAB) or sham surgery. Three weeks later, all animals were subjected to an echocardiographic (echo) evaluation. The following day, either the formation of mitochondrial reactive oxygen species (ROS) or cardiomyocyte (CM) function was examined. The mice were further subjected to hemodynamic (hemo) measurements with subsequent determination of the ventricular weights.

Analgesic buprenorphine hydrochloride (Temgesic^®^, 0.1 mg/kg, Sigma-Aldrich, Steinheim, Germany) was given subcutaneously 30 min prior to surgery. Anaesthesia was initiated by isoflurane 3–4% and maintained by isoflurane 1.5–2.5% supplemented with 100% oxygen. Afterwards, mice were intubated and mechanically ventilated using a mouse ventilator MiniVent type 845 (Hugo Sachs Elektronik, March-Hugstetten, Germany), while placed on a heating surface. After left anterolateral thoracotomy via the second intercostal space, a small titanium clip (Hemoclip^®^, Edward Weck & Co., Inc., Research Triangle Park, NC, USA) was placed around the pulmonary trunk with a specially modified hemoclip applier in order to produce a 65–70% constriction of the pulmonary artery. The chest and afterwards the skin were closed with 6.0 prolene sutures. Sham operated mice underwent the same procedure without applying the hemoclip. In total, 31 WT and 33 p66KO mice were subjected to surgery for this study. The design of the study is shown in Figure 4.

### 5.3. Echocardiography

Vevo2100 high-resolution imaging system equipped by 30-MHz transducer (VisualSonics, Toronto, ON, Canada) was used to perform transthoracic echocardiography three weeks after operation. Anaesthesia was initiated with 3–4% isoflurane and maintained with 2–3% isoflurane in oxygen. For in vivo heart function evaluation, the right ventricular wall thickness, right ventricular internal diameter, tricuspid annular plane systolic excursion, left ventricular end-diastolic internal diameter, left ventricular end-systolic diameter, end-diastolic interventricular septum wall thickness, and end-diastolic left ventricular posterior wall thickness, fractional shortening of the left ventricle, and cardiac index were measured as described before [50,58]. Heart rate was monitored by taping all legs to ECG electrodes. Calculations were performed offline with the software Vevo LAB.

### 5.4. Invasive Hemodynamic Measurement

Hemodynamic measurements were conducted under anesthesia three weeks after PAB or sham operation.

The animals were anaesthetized with 3–4% isoflurane in oxygen and ventilated with a rodent ventilator (Harvard Apparatus, Holiston, MA, USA). Maintenance of anesthesia was done with 2–3% isoflurane supplemented with oxygen. The mice were laid supine on a heating platform with three legs taped to electrocardiogram electrodes for monitoring of heart rate. A rectal thermometer (Indus Instruments, Houston, TX, USA) was used to control the body temperature. The heating pad helped to keep the body temperature at 36.5–37.5 °C. Invasive hemodynamic measurements were performed using a micro pressure catheter (Millar instruments, Houston, TX, USA). The right jugular vein was cannulated for measurement of RV systolic pressure. Systemic arterial pressure was measured via the right carotid artery.

### 5.5. RV Hypertrophy Assessment

Separation of right ventricular wall from left ventricular wall and ventricular septum was done. After heart hypertrophy measurements, the right ventricular wall, left ventricular wall, and septum were fixed in formalin (3.5–3.7%), dehydrated, and finally paraffin embedded to determine dry weights.

### 5.6. Isolation and Culture of Adult Mouse Ventricular Cardiomyocytes

Right ventricular cardiomyocytes were isolated separately from WT and p66KO mice as described in detail elsewhere [59]. After deep anesthesia with isoflurane (4–5%), hearts were excised from the chest cavity, transferred rapidly to ice-cold saline, and immediately mounted on the cannula of Langendorff perfusion system. Heart perfusion and next steps were all done at 37 °C. Perfusion of the heart was performed with the non-circulating mode until 5 mL of perfusate passed the heart (perfusate in mmol/L: NaCl 110, KH_2_PO_4_ 1.2, KCl 2.5, MgSO_4_ 1.2, NaHCO_3_ 25, glucose 10). Afterwards, perfusion was continued with recirculation of 50 mL of the perfusate supplemented with 20 mg collagenase and 25 μM CaCl_2_ with a drop velocity of 1 drop/s. Ventricular tissue was minced after 25 min and incubated in 5mL of the recirculating medium for 5 min. The prepared suspension was filtered through a 200 μm nylon mesh, washed four times following centrifugation (1 min, 25 g) and resuspended in the perfusate, in which the concentration of CaCl_2_ was increased gradually (125 μM, 250 μM, 500 μM, and 1000 μM). Afterwards, the cell pellet was resuspended in serum-free culture medium (medium 199 with Earle’s salts, 15 mM HEPES, 5 mM creatine, 2 mM L-carnitine, 5 mM taurine, 100 IU/mL penicillin, and 100 μg/mL streptomycin). Cells were plated on 35 mm cell culture dishes (Falcon, type 3001) which were pre-coated with laminin (5 μg/mL). After 45 min, cell cultures were washed with the serum free medium to remove non-attached and round cells. Cells were used within the next two hours.

### 5.7. Load-Free Cell Shortening

Cell contraction determination was carried out at room temperature and analyzed using a cell-edge-detection system as previously described [60]. Stimulation of cells was done via two AgCl electrodes with biphasic electrical stimuli made of two equal but opposite rectangular 50 V stimuli of 0.5 ms duration. A voltage of 2 Hz for a duration of 1 min was chosen for the stimulation of each cell. Contractions were recorded after every 15 s. The mean of four measurements at a given frequency was used to calculate the cell shortening of each cell. A line camera (data recording at 500 Hz) was used to measure cell lengths. Cells were used in M199 with an extracellular calcium concentration of 1.25 mM.

### 5.8. Isolation of Mitochondria

To study ROS formation, subsarcolemmal (SSM) mitochondria were isolated from right ventricles. The isolation protocol is based on the previously described protocol of Boengler et al., 2009 [61]. All steps were performed at 4 °C. Right ventricles were washed in buffer A (100 mM KCl, 50 mM 3-[N-Morpholino]-propanesulfonic acid (MOPS), 5 mM MgSO_4_, 1 mM ATP, 1 mM EGTA, pH 7.4), weighed, the tissue was minced in a drop of buffer A on a slide using a scalpel and was then disrupted with a Potter-Elvejhem tissue homogenizer. The homogenate was centrifuged for 10 min at 800 g. The resulting supernatant, which contained the SSM, was centrifuged for 10 min at 8000× *g*. The sedimented mitochondria were washed in buffer A and were resuspended in a small volume of buffer A without ATP.

### 5.9. Reactive Oxygen Species (ROS) Formation

ROS formation was measured as described previously [31]. Twentyfive microgram of isolated mitochondria were transferred to incubation buffer (125 mM KCl, 10 mM Tris-MOPS, 1.2 mM MgCl_2_, 1.2 mM KH_2_PO_4_, 20 μM EGTA, pH 7.4) supplemented with 5 mM glutamate and 2.5 mM malate, 50 μM Amplex UltraRed (Invitrogen, Eugene, OR, USA), and 0.1 U/mL horseradish peroxidase. The fluorescence was measured continuously for 5 min with a Cary Eclipse spectrophotometer (Agilent Technologies, Santa Clara, CA, USA) at the excitation/emission wavelengths of 565/581 nm, respectively. As positive control served control mitochondria supplemented with 6.25 μM of the complex I inhibitor rotenone. Background fluorescence of the buffer without mitochondria was subtracted and the slope of the increase of fluorescence in arbitrary units/time (1.5 min) was calculated.

### 5.10. Western Blot Analysis

Left ventricular tissue sections were lysed in 1 × Cell Lysis buffer (25 mM Tris, 150 mM NaCl, 1 mM EDTA, 1% NP-40, 5% glycerol, pH 7.4) supplemented with 1X PhosStop and Complete inhibitors (Roche, Basel, Switzerland) as well as 1 μM neocuproine. Protein concentration was determined using the Lowry assay. Ten microgram proteins were electrophoretically separated on 10% Bis/Tris gels and proteins were transferred to nitrocellulose membranes. After staining membranes with Ponceau S, membranes were blocked and incubated with rabbit polyclonal anti-human/rat SHC antibodies (BD Biosciences). After washing and incubation with the respective secondary antibodies, immunoreactive signals were detected by chemiluminescence (SuperSignal West Femto or SuperSignal West Pico Chemiluminescent Substrate, ThermoFisher, Waltham, MA, USA).

### 5.11. Statistical Analysis

Data presented in the figures were expressed as boxplots with 25%, 50%, and 75% quartiles, with whiskers representing the total range and with dots represent the individual data points. Data presented in the tables were expressed as means ± SD. A *p*-value < 0.05 is considered to indicate a significant difference. Data were analyzed by two-way ANOVA, following Bonferroni corrections or by t-test. The program SigmaStat 3.5 (Systat, Software GmbH, Erkrath, Germany) was used for statistical analysis.

## 6. Conclusions

Taken together, we demonstrated that mitochondrial ROS formation was unaffected by p66shc deletion following three weeks of pressure overload. Accordingly, cell and heart function of p66KO animals were not different from that of WT animals three weeks after PAB and we conclude that p66shc is not involved in the development and progression of RVH induced by PAB surgery.

## Figures and Tables

**Table 1:** Cell shortening of isolated right ventricular (RV) cardiomyocytes three weeks after pulmonary artery banding (PAB) or sham surgery. Data show the diastolic cell length (Ldiast in µm), contraction velocity (Con Vel in µm/s), relaxation velocity (Rel Vel in µm/s) and load-free cell shortening (dL/L in %) of isolated RV myocytes. Data are given for wild type (WT) and p66shc knockout (p66KO) mice three weeks after sham surgery or PAB. Data are shown as means ± SD from 101 cells (WT sham), 153 cells (p66KO sham), 106 cells (WT PAB) and 131 cells (p66KO PAB), respectively (4 mice each group). #: *p* < 0.05 vs. WT PAB as analyzed by two-way ANOVA.

RV	Ldiast [µm]	Con Vel [µm/s]	Rel Vel [µm/s]	dL/L [%]
sham WT	115.5 ± 6.8	265 ± 59	207 ± 46	10.7 ± 2.0
sham p66KO	111.6 ± 8.9	259 ± 34	242 ± 32	9.7 ± 1.2
PAB WT	113.2 ± 9.6	288 ± 67	240 ± 61	11.3 ± 1.8
PAB p66KO	113.7 ± 8.6	256 ± 80	255 ± 92	9.4 ± 2.1 **#**

**Table 2:** Body weight (BW) and hemodynamic values in mice three weeks after pulmonary artery banding (PAB) or sham surgery. Data show the BW (in g), heart rate (HR in bpm) and systemic arterial pressure (SAP in mmHg) of wild type (WT) and p66shc knockout (p66KO) mice three weeks after sham surgery or PAB. Data are shown as means ± SD. Data for BW and HR were pooled from the animals of all study groups (WT sham: *n* = 16, WT PAB: *n* = 15, p66KO sham: *n* = 15 and p66KO PAB: *n* = 18, respectively). Data for SAP were obtained from the animals included in hemodynamic analysis (WT sham: *n* = 6, WT PAB: *n* = 7, p66KO sham: *n* = 6 and p66KO PAB: *n* = 9, respectively). Data were compared by two-way ANOVA.

RV	BW [g]	HR [bpm]	SAP [mmHg]
sham WT	28.6 ± 3.3	513 ± 45	73.7 ± 8.3
sham p66KO	27.2 ± 3.2	486 ± 57	65.5 ± 15.5
PAB WT	28.9 ± 3.1	502 ± 48	71.5 ± 9.5
PAB p66KO	26.6 ± 2.2	470 ± 50	67.7 ± 12.5

**Table 3:** Right ventricular weight and ventricular geometry in mice three weeks after pulmonary artery banding (PAB) or sham surgery. Data show the right ventricular weight normalized to body weight (RV/BW in mg/g), the right ventricular wall thickness (RVWT in mm) and the right ventricular inner diameter (RVID in mm). Data are given for wild type (WT) and p66shc knockout (p66KO) mice three weeks after sham surgery (*n* = 6 each group) and for WT and p66KO mice three weeks after PAB (*n* = 7 and *n* = 9, respectively). Data are shown as means ± SD. *: *p* < 0.05 vs. WT sham and **: *p* < 0.05 vs. p66KO sham as analyzed by two-way ANOVA.

RV	RV/BW [mg/g]	RVWT [mm]	RVID [mm]
sham WT	0.9 ± 0.1	0.21 ± 0.01	1.03 ± 0.09
sham p66KO	0.8 ± 0.1	0.20 ± 0.01	1.04 ± 0.08
PAB WT	1.5 ± 0.2 *	0.42 ± 0.05 *	2.03 ± 0.14 *
PAB p66KO	1.3 ± 0.3 **	0.41 ± 0.03 **	1.94 ± 0.28 **

## Supplementary Materials

Supplementary Materials can be found at https://www.mdpi.com/1422-0067/21/24/9339/s1.

## Abbreviations

Ang II	angiotensin II
BW	body weight
CM	cardiomyocyte
CI	cardiac index
Con Vel	contraction velocity
dL/L	load-free cell shortening
echo	echocardiographic
hemo	hemodynamic
HR	heart rate
Ldiast	diastolic cell length
LV	left ventricle
MAP	mitogen activated protein
MCT	monocrotaline
NADPH	nicotinamide adenine dinucleotide phosphate
p66KO	p66shc knockout
PAB	pulmonary artery banding
PAH	pulmonary arterial hypertension
Rel Vel	relaxation velocity
ROS	reactive oxygen species
RV	right ventricle
RV/(LV + S)	right ventricular weight normalized to the left ventricle and septum weight
RV/BW	right ventricular weight normalized to body weight
RVF	right ventricular failure
RVH	right ventricular hypertrophy
RVID	right ventricular inner diameter
RVSP	right ventricular systolic pressure
RVWT	right ventricular wall thickness
RyR2	cardiac ryanodine receptor
SAP	systemic arterial pressure
SD	standard deviation
TAC	transverse aortic constriction
TAPSE	tricuspid annular plane systolic excursion
MnSOD	manganese superoxide dismutase
NADPH	nicotinamide adenine dinucleotide phosphate
ETC	electron transport chain
shc	spontaneous human combustion
SERCA	sarcoplasmic reticulum calcium ATPase
WT	wild type

## References

[1] Ryan J.J., Archer S.L. (2014). The right ventricle in pulmonary arterial hypertension: Disorders of metabolism, angiogenesis and adrenergic signaling in right ventricular failure. Circ. Res..

[2] Ecarnot-Laubriet A., Rochette L., Vergely C., Sicard P., Teyssier J.R. (2003). The activation pattern of the antioxidant enzymes in the right ventricle of rat in response to pressure overload is of heart failure type. Heart Dis..

[3] Farahmand F., Hill M.F., Singal P.K. (2004). Antioxidant and oxidative stress changes in experimental cor pulmonale. Mol. Cell. Biochem..

[4] Hansen T., Galougahi K.K., Celermajer D., Rasko N., Tang O., Bubb K.J., Figtree G. (2016). Oxidative and nitrosative signalling in pulmonary arterial hypertension—Implications for development of novel therapies. Pharmacol. Ther..

[5] Schluter K.D., Kutsche H.S., Hirschhauser C., Schreckenberg R., Schulz R. (2018). Review on Chamber-Specific Differences in Right and Left Heart Reactive Oxygen Species Handling. Front. Physiol..

[6] Redout E.M., Wagner M.J., Zuidwijk M.J., Boer C., Musters R.J., van Hardeveld C., Paulus W.J., Simonides W.S. (2007). Right-ventricular failure is associated with increased mitochondrial complex II activity and production of reactive oxygen species. Cardiovasc. Res..

[7] Sabri A., Hughie H.H., Lucchesi P.A. (2003). Regulation of hypertrophic and apoptotic signaling pathways by reactive oxygen species in cardiac myocytes. Antioxid. Redox Signal..

[8] Di Lisa F., Kaludercic N., Carpi A., Menabo R., Giorgio M. (2009). Mitochondrial pathways for ROS formation and myocardial injury: The relevance of p66(Shc) and monoamine oxidase. Basic Res. Cardiol..

[9] Di Lisa F., Giorgio M., Ferdinandy P., Schulz R. (2017). New aspects of p66Shc in ischaemia reperfusion injury and other cardiovascular diseases. Br. J. Pharmacol..

[10] Di Lisa F., Kaludercic N., Paolocci N. (2011). β2-Adrenoceptors, NADPH oxidase, ROS and p38 MAPK: Another ‘radical’ road to heart failure?. Br. J. Pharmacol..

[11] Date M.O., Morita T., Yamashita N., Nishida K., Yamaguchi O., Higuchi Y., Hirotani S., Matsumura Y., Hori M., Tada M. (2002). The antioxidant N-2-mercaptopropionyl glycine attenuates left ventricular hypertrophy in in vivo murine pressure-overload model. J. Am. Coll. Cardiol..

[12] Dhalla A.K., Hill M.F., Singal P.K. (1996). Role of oxidative stress in transition of hypertrophy to heart failure. J. Am. Coll. Cardiol..

[13] van Empel V.P., Bertrand A.T., van Oort R.J., van der Nagel R., Engelen M., van Rijen H.V., Doevendans P.A., Crijns H.J., Ackerman S.L., Sluiter W. (2006). EUK-8, a superoxide dismutase and catalase mimetic, reduces cardiac oxidative stress and ameliorates pressure overload-induced heart failure in the harlequin mouse mutant. J. Am. Coll. Cardiol..

[14] Pinton P., Rimessi A., Marchi S., Orsini F., Migliaccio E., Giorgio M., Contursi C., Minucci S., Mantovani F., Wieckowski M.R. (2007). Protein kinase C beta and prolyl isomerase 1 regulate mitochondrial effects of the life-span determinant p66Shc. Science.

[15] Le S., Connors T.J., Maroney A.C. (2001). c-Jun N-terminal kinase specifically phosphorylates p66ShcA at serine 36 in response to ultraviolet irradiation. J. Biol. Chem..

[16] Giorgio M., Migliaccio E., Orsini F., Paolucci D., Moroni M., Contursi C., Pelliccia G., Luzi L., Minucci S., Marcaccio M. (2005). Electron transfer between cytochrome c and p66Shc generates reactive oxygen species that trigger mitochondrial apoptosis. Cell.

[17] Smith W.W., Norton D.D., Gorospe M., Jiang H., Nemoto S., Holbrook N.J., Finkel T., Kusiak J.W. (2005). Phosphorylation of p66Shc and forkhead proteins mediates Aβ toxicity. J. Cell Biol..

[18] Lebiedzinska M., Karkucinska-Wieckowska A., Giorgi C., Karczmarewicz E., Pronicka E., Pinton P., Duszynski J., Pronicki M., Wieckowski M.R. (2010). Oxidative stress-dependent p66Shc phosphorylation in skin fibroblasts of children with mitochondrial disorders. Biochim. Biophys. Acta.

[19] Galimov E.R. (2010). The Role of p66shc in Oxidative Stress and Apoptosis. Acta Nat..

[20] Oshikawa J., Kim S.J., Furuta E., Caliceti C., Chen G.F., McKinney R.D., Kuhr F., Levitan I., Fukai T., Ushio-Fukai M. (2012). Novel role of p66Shc in ROS-dependent VEGF signaling and angiogenesis in endothelial cells. Am. J. Physiol. Heart Circ. Physiol..

[21] Xiao Y., Xia J., Cheng J., Huang H., Zhou Y., Yang X., Su X., Ke Y., Ling W. (2019). Inhibition of S-Adenosylhomocysteine Hydrolase Induces Endothelial Dysfunction via Epigenetic Regulation of p66shc-Mediated Oxidative Stress Pathway. Circulation.

[22] Carpi A., Menabo R., Kaludercic N., Pelicci P., Di Lisa F., Giorgio M. (2009). The cardioprotective effects elicited by p66(Shc) ablation demonstrate the crucial role of mitochondrial ROS formation in ischemia/reperfusion injury. Biochim. Biophys. Acta.

[23] Migliaccio E., Giorgio M., Mele S., Pelicci G., Reboldi P., Pandolfi P.P., Lanfrancone L., Pelicci P.G. (1999). The p66shc adaptor protein controls oxidative stress response and life span in mammals. Nature.

[24] Obreztchikova M., Elouardighi H., Ho M., Wilson B.A., Gertsberg Z., Steinberg S.F. (2006). Distinct signaling functions for Shc isoforms in the heart. J. Biol. Chem..

[25] Cesselli D., Jakoniuk I., Barlucchi L., Beltrami A.P., Hintze T.H., Nadal-Ginard B., Kajstura J., Leri A., Anversa P. (2001). Oxidative stress-mediated cardiac cell death is a major determinant of ventricular dysfunction and failure in dog dilated cardiomyopathy. Circ. Res..

[26] Graiani G., Lagrasta C., Migliaccio E., Spillmann F., Meloni M., Madeddu P., Quaini F., Padura I.M., Lanfrancone L., Pelicci P. (2005). Genetic deletion of the p66Shc adaptor protein protects from angiotensin II-induced myocardial damage. Hypertension.

[27] Schreckenberg R., Rebelo M., Deten A., Weber M., Rohrbach S., Pipicz M., Csonka C., Ferdinandy P., Schulz R., Schluter K.D. (2015). Specific Mechanisms Underlying Right Heart Failure: The Missing Upregulation of Superoxide Dismutase-2 and Its Decisive Role in Antioxidative Defense. Antioxid. Redox Signal..

[28] Luitel H., Sydykov A., Schymura Y., Mamazhakypov A., Janssen W., Pradhan K., Wietelmann A., Kosanovic D., Dahal B.K., Weissmann N. (2017). Pressure overload leads to an increased accumulation and activity of mast cells in the right ventricle. Physiol. Rep..

[29] Boengler K., Bornbaum J., Schluter K.D., Schulz R. (2019). P66shc and its role in ischemic cardiovascular diseases. Basic Res. Cardiol..

[30] Trinei M., Giorgio M., Cicalese A., Barozzi S., Ventura A., Migliaccio E., Milia E., Padura I.M., Raker V.A., Maccarana M. (2002). A p53-p66Shc signalling pathway controls intracellular redox status, levels of oxidation-damaged DNA and oxidative stress-induced apoptosis. Oncogene.

[31] Galimov E.R., Chernyak B.V., Sidorenko A.S., Tereshkova A.V., Chumakov P.M. (2014). Prooxidant properties of p66shc are mediated by mitochondria in human cells. PLoS ONE.

[32] Wang Y., Zhao J., Yang W., Bi Y., Chi J., Tian J., Li W. (2015). High-dose alcohol induces reactive oxygen species-mediated apoptosis via PKC-beta/p66Shc in mouse primary cardiomyocytes. Biochem. Biophys. Res. Commun..

[33] Paulin R., Michelakis E.D. (2014). The metabolic theory of pulmonary arterial hypertension. Circ. Res..

[34] Zimmer A., Teixeira R.B., Bonetto J.H.P., Bahr A.C., Turck P., de Castro A.L., Campos-Carraro C., Visioli F., Fernandes-Piedras T.R., Casali K.R. (2020). Role of inflammation, oxidative stress, and autonomic nervous system activation during the development of right and left cardiac remodeling in experimental pulmonary arterial hypertension. Mol. Cell. Biochem..

[35] Frazziano G., Al Ghouleh I., Baust J., Shiva S., Champion H.C., Pagano P.J. (2014). Nox-derived ROS are acutely activated in pressure overload pulmonary hypertension: Indications for a seminal role for mitochondrial Nox4. Am. J. Physiol. Heart Circ. Physiol..

[36] Qipshidze N., Tyagi N., Metreveli N., Lominadze D., Tyagi S.C. (2012). Autophagy mechanism of right ventricular remodeling in murine model of pulmonary artery constriction. Am. J. Physiol. Heart Circ. Physiol..

[37] Pak O., Scheibe S., Esfandiary A., Gierhardt M., Sydykov A., Logan A., Fysikopoulos A., Veit F., Hecker M., Kroschel F. (2018). Impact of the mitochondria-targeted antioxidant MitoQ on hypoxia-induced pulmonary hypertension. Eur. Respir. J..

[38] Li N., Ragheb K., Lawler G., Sturgis J., Rajwa B., Melendez J.A., Robinson J.P. (2003). Mitochondrial complex I inhibitor rotenone induces apoptosis through enhancing mitochondrial reactive oxygen species production. J. Biol. Chem..

[39] Orsini F., Moroni M., Contursi C., Yano M., Pelicci P., Giorgio M., Migliaccio E. (2006). Regulatory effects of the mitochondrial energetic status on mitochondrial p66Shc. Biol. Chem..

[40] Guo J., Gertsberg Z., Ozgen N., Steinberg S.F. (2009). p66Shc links alpha1-adrenergic receptors to a reactive oxygen species-dependent AKT-FOXO3A phosphorylation pathway in cardiomyocytes. Circ. Res..

[41] Sharma K., Kass D.A. (2014). Heart failure with preserved ejection fraction: Mechanisms, clinical features, and therapies. Circ. Res..

[42] Spruijt O.A., de Man F.S., Groepenhoff H., Oosterveer F., Westerhof N., Vonk-Noordegraaf A., Bogaard H.-J. (2015). The Effects of Exercise on Right Ventricular Contractility and Right Ventricular–Arterial Coupling in Pulmonary Hypertension. Am. J. Respir. Crit. Care Med..

[43] Wang Z., Schreier D.A., Hacker T.A., Chesler N.C. (2013). Progressive right ventricular functional and structural changes in a mouse model of pulmonary arterial hypertension. Physiol. Rep..

[44] Esfandiary A., Kutsche H.S., Schreckenberg R., Weber M., Pak O., Kojonazarov B., Sydykov A., Hirschhauser C., Wolf A., Haag D. (2019). Protection against pressure overload-induced right heart failure by uncoupling protein 2 silencing. Cardiovasc. Res..

[45] Santos C.X., Anilkumar N., Zhang M., Brewer A.C., Shah A.M. (2011). Redox signaling in cardiac myocytes. Free Radic. Biol. Med..

[46] Baysa A., Sagave J., Carpi A., Zaglia T., Campesan M., Dahl C.P., Bilbija D., Troitskaya M., Gullestad L., Giorgio M. (2015). The p66ShcA adaptor protein regulates healing after myocardial infarction. Basic Res. Cardiol..

[47] Rota M., LeCapitaine N., Hosoda T., Boni A., De Angelis A., Padin-Iruegas M.E., Esposito G., Vitale S., Urbanek K., Casarsa C. (2006). Diabetes promotes cardiac stem cell aging and heart failure, which are prevented by deletion of the p66shc gene. Circ. Res..

[48] Holda M.K., Klimek-Piotrowska W., Koziej M., Piatek K., Holda J. (2016). Influence of different fixation protocols on the preservation and dimensions of cardiac tissue. J. Anat..

[49] Sheikh F., Raskin A., Chu P.H., Lange S., Domenighetti A.A., Zheng M., Liang X., Zhang T., Yajima T., Gu Y. (2008). An FHL1-containing complex within the cardiomyocyte sarcomere mediates hypertrophic biomechanical stress responses in mice. J. Clin. Investig..

[50] Veith C., Neghabian D., Luitel H., Wilhelm J., Egemnazarov B., Muntanjohl C., Fischer J.H., Dahal B.K., Schermuly R.T., Ghofrani H.A. (2020). FHL-1 is not involved in pressure overload-induced maladaptive right ventricular remodeling and dysfunction. Basic Res. Cardiol..

[51] Bokma J.P., Winter M.M., Dijk A.P.V., Vliegen H.W., Melle J.P.V., Meijboom F.J., Post M.C., Berbee J.K., Boekholdt S.M., Groenink M. (2018). Effect of Losartan on Right Ventricular Dysfunction. Circulation.

[52] Reddy S., Bernstein D., Newburger J.W. (2018). Renin-Angiotensin-Aldosterone System Inhibitors for Right Ventricular Dysfunction in Tetralogy of Fallot. Circulation.

[53] Boehm M., Lawrie A., Wilhelm J., Ghofrani H.A., Grimminger F., Weissmann N., Seeger W., Schermuly R.T., Kojonazarov B. (2017). Maintained right ventricular pressure overload induces ventricular-arterial decoupling in mice. Exp. Physiol..

[54] Gierhardt M., Pak O., Sydykov A., Kraut S., Schäffer J., Garcia C., Veith C., Zeidan E.M., Brosien M., Quanz K. Genetic deletion of p66shc and/or cyclophilin D results in decreased pulmonary vascular tone. Cardiovasc. Res..

[55] Boengler K., Bencsik P., Paloczi J., Kiss K., Pipicz M., Pipis J., Ferdinandy P., Schluter K.D., Schulz R. (2017). Lack of Contribution of p66shc and Its Mitochondrial Translocation to Ischemia-Reperfusion Injury and Cardioprotection by Ischemic Preconditioning. Front. Physiol..

[56] Lone A., Harris R.A., Singh O., Betts D.H., Cumming R.C. (2018). p66Shc activation promotes increased oxidative phosphorylation and renders CNS cells more vulnerable to amyloid beta toxicity. Sci. Rep..

[57] Edwards N.A., Watson A.J., Betts D.H. (2016). P66Shc, a key regulator of metabolism and mitochondrial ROS production, is dysregulated by mouse embryo culture. Mol. Hum. Reprod..

[58] Pullamsetti S.S., Kojonazarov B., Storn S., Gall H., Salazar Y., Wolf J., Weigert A., El-Nikhely N., Ghofrani H.A., Krombach G.A. (2017). Lung cancer-associated pulmonary hypertension: Role of microenvironmental inflammation based on tumor cell-immune cell cross-talk. Sci. Transl. Med..

[59] Botker H.E., Hausenloy D., Andreadou I., Antonucci S., Boengler K., Davidson S.M., Deshwal S., Devaux Y., Di Lisa F., Di Sante M. (2018). Practical guidelines for rigor and reproducibility in preclinical and clinical studies on cardioprotection. Basic Res. Cardiol..

[60] Langer M., Luttecke D., Schluter K.D. (2003). Mechanism of the positive contractile effect of nitric oxide on rat ventricular cardiomyocytes with positive force/frequency relationship. Pflug. Arch. Eur. J. Physiol..

[61] Boengler K., Stahlhofen S., van de Sand A., Gres P., Ruiz-Meana M., Garcia-Dorado D., Heusch G., Schulz R. (2009). Presence of connexin 43 in subsarcolemmal, but not in interfibrillar cardiomyocyte mitochondria. Basic Res. Cardiol..

