# Supplementary material for: Lack of Contribution of p66shc to Pressure Overload-Induced Right Heart Hypertrophy
Source: Int J Mol Sci. 2020 Dec 8;21(24):9339. doi: 10.3390/ijms21249339 (PMC7762598; doi:10.3390/ijms21249339)
Supplement: Supplementary file 1 [file ijms-21-09339-s001.pdf]

Supplement

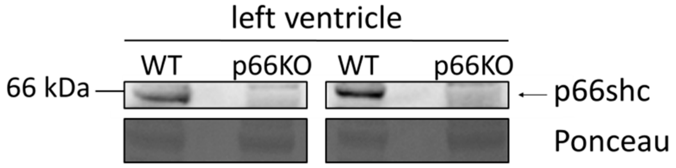

Figure 1: p66shc protein expression in left ventricular myocardium. Western Blot analysis was performed for p66shc on left ventricular (LV) tissue samples obtained from wild type (WT, n=4) and p66shc knockout (p66KO, n=4) mice. Ponceau S staining is shown to demonstrate equal protein loading.

Table 1: Cardiac function in mice 3 weeks after pulmonary artery banding or sham surgery which were included in the analysis of mitochondrial ROS formation. Data show the right ventricular wall thickness (RVWT in mm), the tricuspid annular plane systolic excursion (TAPSE, in mm), and cardiac index (CI, in ml/min/g BW). Data are given for wild type (WT) and p66shc knockout (p66KO) mice 3 weeks after sham surgery (n=4 and n=5, respectively) and for WT and p66KO mice 3 weeks after pulmonary artery banding (PAB, n=6 and n=5, respectively). Data are expressed as means ± SD from four mice each group. *: p<0.05 vs. WT sham and **: p<0.05 vs. p66KO sham as analyzed by two-way ANOVA.

RV	RVWT [mm]	TAPSE [mm]	CI [ml/min/g BW]
Sham WT	0.22 ± 0.003	1.26 ± 0.02	0.79 ± 0.02
Sham p66KO	0.22 ± 0.01	1.26 ± 0.04	0.79 ± 0.07
PAB WT	0.48 ± 0.01*	0.75 ± 0.06*	0.58 ± 0.2 *
PAB p66KO	0.49 ± 0.03**	0.73 ± 0.04**	0.52 ± 0.15**

Table 2: Cardiac function in mice 3 weeks after pulmonary artery banding or sham surgery which were included in cardiomyocyte function analysis. Data show the right ventricular wall thickness (RVWT in mm), the tricuspid annular plane systolic excursion (TAPSE, in mm), and cardiac index (CI, in ml/min/g BW). Data are given for wild type (WT) and p66shc knockout (p66KO) mice 3 weeks after sham surgery (n=4 and n=4, respectively) and for WT and p66KO mice 3 weeks after pulmonary artery banding (PAB, n=4 and n=4, respectively). Data are expressed as means ± SD from. *:p<0.05 vs. WT sham and **: p<0.05 vs. p66KO sham as analyzed by two-way ANOVA.

RV	RVWT [mm]	TAPSE [mm]	CI [ml/min/g BW]
Sham WT	0.23 ± 0.014	1.09 ± 0.2	0.84 ± 0.1
Sham p66KO	0.22 ± 0.003	1.18 ± 0.1	0.78 ± 0.2
PAB WT	0.48 ± 0.041*	0.73 ± 0.1*	0.63 ± 0.1*
PAB p66KO	0.48 ± 0.016**	0.77 ± 0.1**	0.50 ± 0.1**

Table 3: Cardiac function in mice 3 weeks after pulmonary artery banding or sham surgery optained from the animals of all study groups. Data show the right ventricular wall thickness (RVWT in mm), the tricuspid annular plane systolic excursion (TAPSE, in mm), and cardiac index (CI, in ml/min/g BW). Data are given for wild type (WT) and p66shc knockout (p66KO) mice 3 weeks after sham surgery (n=16 and n=15, respectively) and for WT and p66KO mice 3 weeks after pulmonary artery banding (PAB, n=15 and n=18, respectively). Data are expressed as means ± SD. *:p<0.05 vs. WT sham and**: p<0.05 vs. p66KO sham as analyzed by two-way ANOVA.

RV	RVWT [mm]	TAPSE [mm]	CI [ml/min/g BW]
Sham WT	0.22 ± 0.02	1.21 ± 0.1	0.80 ± 0.1
Sham p66KO	0.21 ± 0.01	1.25 ± 0.1	0.79 ± 0.1
PAB WT	0.46 ± 0.04*	0.79 ± 0.1*	0.59 ± 0.1*
PAB p66KO	0.45 ± 0.04**	0.82 ± 0.1**	0.55 ± 0.1**

Table 4: Echocardiographic parameters of left ventricular structure and systolic function obtained from mice 3 weeks after sham surgery. Data show the left ventricular (LV) mass in mg, the LV end-diastolic interventricular septum thickness (IVSd, in mm), the LV internal diameter at end-diastole (LVIDd, in mm), the LV internal diameter at end-systole (LVIDs, in mm), the LV posterior wall thickness at end-diastole (LVPWd, in mm), the fractional shortening (FS, in %) and the cardiac index (CI, in ml/min/g BW). Data are given for sham surgery of wild type (WT, n=6) and p66shc knockout (p66KO, n=6) mice and are shown as means +/- SD. *: $p<0.05$ vs. WT sham as analyzed by t-test.

**LV**	**WT**	**p66KO**
Mass (mg)	92.9 ± 11	98 ± 15,85
IVSd (mm)	0.7 ± 0.1	0.65 ± 0.1
LVIDd (mm)	3.9 ± 0.2	4.2 ± 0.3
LVIDs (mm)	2.6 ± 0.2	3.0 ± 0.3 *
LVPWd (mm)	0.7 ± 0.1	0.65 ± 0.1
FS (%)	33.2 ± 3.0	27.8 ± 2.5*
CI (ml/min/g BW)	0.83 ± 0.1	0.82 ± 0.1
